# Different Approaches to Extracting Proximally Migrated or Broken and Retained Pancreatic Stents

**DOI:** 10.3390/jcm14124298

**Published:** 2025-06-17

**Authors:** Navkiran Randhawa, Ahamed Khalyfa, Raahi Patel, Rahil Desai, Mahnoor Inamullah, Haoran Peng, Varshita Goduguchinta, Subbaramiah Sridhar, Kamran Ayub

**Affiliations:** 1 Division of Gastroenterology and Hepatology, Augusta University, Augusta, GA 30912, USA; nrandhawa@augusta.edu (N.R.); hpeng@augusta.edu (H.P.); ssridhar@augusta.edu (S.S.); 2 Department of Gastroenterology, University of Iowa, Iowa City, IA 52242, USA; akhalyfa1@gmail.com; 3 Franciscan Health Olympia Fields, Olympia Fields, IL 60461, USA; raahip07@gmail.com (R.P.); rahildesai@gmail.com (R.D.); vogoduguchinta1@gmail.com (V.G.); 4 Southwest Gastroenterology, Oak Lawn, IL 60453, USA; mahnoor.fm93@gmail.com; 5 Silver Cross Hospital, New Lenox, IL 60451, USA

**Keywords:** pancreatic stents, stent migration, endoscopic retrieval, ERCP, SpyGlass system, balloon sweep, Soehendra retriever, pancreatic duct obstruction, endoscopic techniques, minimally invasive therapy

## Abstract

**Background:** Pancreatic stents (PSs) play a crucial role in the management of pancreatic duct obstructions, particularly in the context of endoscopic retrograde cholangiopancreatography (ERCP). However, stent migration remains a significant complication, leading to risks such as pancreatitis, pancreatic duct stenosis, and abscess formation. This study aims to evaluate the efficacy of various endoscopic techniques for retrieving proximally migrated or broken pancreatic stents, highlighting optimal strategies for improving patient outcomes. **Methods:** A retrospective multicenter review was conducted across six hospitals from 2016 to 2024. Patients with proximally migrated or broken pancreatic stents referred for endoscopic retrieval after failed attempts at other facilities were included. Demographic data, stent characteristics, and retrieval techniques were analyzed. Endoscopic methods included SpyGlass forceps, SpyGlass baskets, Soehendra retriever stents, balloon sweeps, flower baskets, and extension pancreatic sphincterotomy. Procedural success, retrieval times, and post-procedural outcomes were assessed. **Results:** Twelve patients underwent endoscopic retrieval, including two with broken stents. All procedures were successful, with retrieval times averaging 30 to 45 min. Two patients developed pancreatic duct narrowing, requiring balloon dilation. All patients had new stents placed to maintain duct patency, and no major complications were observed. Follow-up evaluations confirmed complete resolution of migration-related issues, with all stents removed. **Conclusions:** Endoscopic retrieval of migrated pancreatic stents is highly effective, with specialized techniques ensuring a 100% success rate in this study. Early intervention and the selection of appropriate retrieval methods are critical in minimizing complications. Further research is needed to refine retrieval strategies and standardize protocols to enhance clinical outcomes.

## 1. Introduction

Pancreatic ductal pathologies, including chronic pancreatitis, pancreatic neoplasms, ductal disruptions, and postoperative pancreatic leaks, represent complex clinical scenarios that often require multidisciplinary interventions. Among the various tools available, pancreatic stents (PSs) have emerged as one of the most indispensable components in the management of these conditions in procedures performed through endoscopic retrograde cholangiopancreatography (ERCP). Initially introduced for the decompression of obstructed ducts and prevention of post-ERCP pancreatitis, pancreatic stents have since evolved into a versatile therapeutic modality utilized across a wide spectrum of indications [1,2].

The primary objective of pancreatic stent placement is to facilitate ductal drainage and reduce intraductal hypertension, and thereby alleviate pain or inflammation, particularly in the setting of chronic pancreatitis or pancreatic duct strictures. In cases involving traumatic or iatrogenic ductal injuries, stents help bridge the disrupted duct and promote healing [3,4]. Moreover, in recent years, the prophylactic use of pancreatic stents has been advocated in high-risk ERCP procedures to mitigate the incidence of post-procedural pancreatitis (PEP), which remains one of the most feared complications of ERCP [5,6]. A meta-analysis involving eight different studies showed that pancreatic stent placement was associated with statistically significant reductions in PEP incidence [5].

With the increasing reliance on PSs for both therapeutic and preventative purposes, technological advancements in stent design have also paralleled clinical demand. Innovations have included the development of variable stent diameters and lengths, incorporation of anti-migration features, and the use of biodegradable materials to reduce the need for subsequent retrieval [7,8]. These design elements aim to optimize ductal patency, reduce tissue irritation, and facilitate spontaneous passage or removal. However, despite such enhancements, the issue of stent migration continues to pose a significant clinical challenge, particularly proximal migration, where the stent moves upstream into the pancreatic ductal system [7,8].

Proximal stent migration, although relatively infrequent with a reported incidence between 2% and 5%, can have serious implications [9]. Some of the associations to proximal stent migration include larger diameter stents, shorter stents, and malignant strictures [10]. The pancreatic duct is narrow, tortuous, and highly sensitive to foreign bodies. When a stent migrates proximally, it can become embedded in the ductal wall, incite an inflammatory response, or even result in ductal perforation or parenchymal injury [11]. Clinically, this can manifest as recurrent pancreatitis, abdominal pain, or the formation of pancreatic pseudocysts and abscesses. Left unaddressed, a migrated stent may lead to progressive ductal stenosis, exacerbating the patient’s underlying disease and, in rare cases, necessitating surgical intervention such as pancreaticojejunostomy or pancreatectomy [12].

Timely retrieval of migrated stents is therefore critical, yet it is often a complex and technically demanding endeavor. Standard endoscopic tools such as snares or biopsy forceps may be insufficient, especially when visualization is limited or the stent has migrated deeply into the pancreatic tail. Factors such as the patient’s ductal anatomy, the presence of strictures, the type and length of stent used, and prior procedural trauma all add layers of complexity to retrieval attempts [13]. Moreover, unsuccessful attempts at retrieval not only prolong the duration of stent retention—thereby increasing complication risk—but also heighten the likelihood of mucosal injury, bleeding, and infection [7,14].

To address these challenges, a range of specialized endoscopic techniques and devices has been utilized. These include the SpyGlass™ Direct Visualization System, which offers direct intraductal visualization to locate and grasp deeply lodged stents; Soehendra stent retrievers, which allow for rotational drilling into embedded stents; flower baskets and rat-tooth forceps designed to improve grip; and balloon-sweep techniques to dislodge stents under fluoroscopic guidance [15,16,17,18]. In particularly difficult cases, techniques such as extension pancreatic sphincterotomy or wire-guided cannulation beyond the stent may be required to access the stent tip or distal flange [19]. While each method has its merits, comparative data on their effectiveness remain limited, particularly in real-world settings involving patients with prior failed retrieval attempts.

The lack of standardized protocols for the retrieval of proximally migrated or fractured pancreatic stents adds further uncertainty to clinical decision-making. Most of the existing literature is limited to single-center retrospective reviews or small case series, offering limited generalizability. Additionally, the heterogeneity in patient anatomy, stent characteristics, operator experience, and device availability across institutions further complicates efforts to establish best practices.

In light of these challenges, there is a pressing need for multicenter data to better understand which techniques yield the highest success rates and lowest complication profiles under diverse clinical circumstances. Our study seeks to fill this gap by offering a comprehensive multicenter retrospective analysis of endoscopic retrieval techniques in cases of proximally migrated or broken pancreatic stents. All patients included in this analysis were referred to our institutions after prior failed retrieval attempts at other healthcare facilities, reflecting a particularly complex patient subset. We evaluated a range of modern retrieval modalities, including SpyGlass-assisted techniques, Soehendra retrievers, balloon sweeps, flower baskets, and extension pancreatic sphincterotomies. By comparing procedural success rates, retrieval times, and post-procedural outcomes across this diverse patient cohort, our study aims to identify the most effective strategies for stent retrieval, with the broader goal of guiding clinical practice, minimizing complications, and improving overall patient outcomes.

In doing so, this research contributes valuable data to an area of gastroenterological intervention that remains underrepresented in the literature. As the use of pancreatic stents continues to expand in scope and frequency, a clearer understanding of how to manage their complications—particularly migration—becomes increasingly important. Ultimately, we aim to develop more robust, evidence-based protocols that can standardize care, reduce variability in outcomes, and ensure safer, more effective interventions for patients with pancreatic stent migration.

## 2. Methods

This retrospective multicenter case series was conducted across six tertiary care hospitals from January 2016 to December 2024. Medical records were reviewed to identify patients with either proximally migrated or broken pancreatic duct stents who were referred for endoscopic retrieval after failed attempts at removal in external facilities. The study was approved by the respective institutional review boards at each participating center. Informed consent for treatment and data usage was obtained at the time of intervention in accordance with ethical standards.

Patients were eligible for inclusion if they presented with a proximally migrated pancreatic stent, defined as a stent that had migrated upstream into the pancreatic duct and could not be accessed or removed with standard retrieval methods, or if the stent had fractured within the duct with retained intraductal components. Only patients with main pancreatic duct (PD) involvement were considered. Patients were excluded if the stent had migrated distally (i.e., toward the duodenum) or if they lacked sufficient follow-up or procedural documentation.

Demographic and clinical data were collected, including patient age, sex, stent characteristics (size, length, and type), duration of stent placement prior to retrieval, and the specific techniques used for stent removal. The stent retrieval methodology was determined by the proceduralist based on the anatomic location of the stent, degree of ductal dilation, presence of strictures, and visibility of the stent under fluoroscopy or direct visualization. Stent removal tools included the SpyGlass™ Direct Visualization System, SpyGlass forceps, snare and basket, Soehendra stent retriever, flower basket, balloon-dilation and sweep techniques, extension pancreatic sphincterotomy, and pediatric or rat-tooth biopsy forceps. A summary of individual patient data is provided in Table 1.

Demographic data, including age, sex, type and size of stents, duration of stent placement, and retrieval techniques, were collected and analyzed (Table 1).

All procedures were performed in the prone position under either monitored anesthesia care (MAC) or general anesthesia, determined by the patient’s clinical condition and anticipated procedural complexity. Of the twelve patients, nine underwent MAC and three required general anesthesia. All ERCPs were performed by two expert interventional endoscopists (K.A. and S.S.) with extensive experience in pancreaticobiliary procedures. Cannulation of the pancreatic duct was accomplished using a traction sphincterotome and guidewire technique. A 0.035-inch guidewire was employed initially in six procedures; in two of these cases, the wire was exchanged for a 0.021-inch guidewire to facilitate access through tight strictures. A 0.025-inch guidewire was used in six patients without requiring exchange. Spyglass was used in patients with pancreatic duct diameter greater than 4 mm.

All the patients received an indomethacin 100 mg rectal suppository at the beginning of the procedure. Intravenous fluid management included administration of lactated Ringer’s solution, both intra-procedurally and post-procedurally, totaling 2 L per patient unless limited by a history of congestive heart failure or volume overload. Eight patients were admitted for overnight observation due to the complexity of the case or baseline comorbidities, while four patients with chronic pancreatitis and dilated ducts were considered low risk for post-ERCP pancreatitis and were safely discharged the same day following anesthesia recovery.

Technical success was defined as the complete endoscopic retrieval of the migrated or fractured stent components without the need for surgical rescue. Procedural time was recorded from initial scope insertion to scope withdrawal. Post-procedural monitoring was conducted for signs of pancreatitis, perforation, bleeding, or infection. Follow-up EUS or, if needed, ERCP was scheduled between 3 and 4 weeks to assess pancreatic ductal integrity and remove any temporary stents placed during the initial retrieval procedure.

## 3. Results

**Figure 1:**
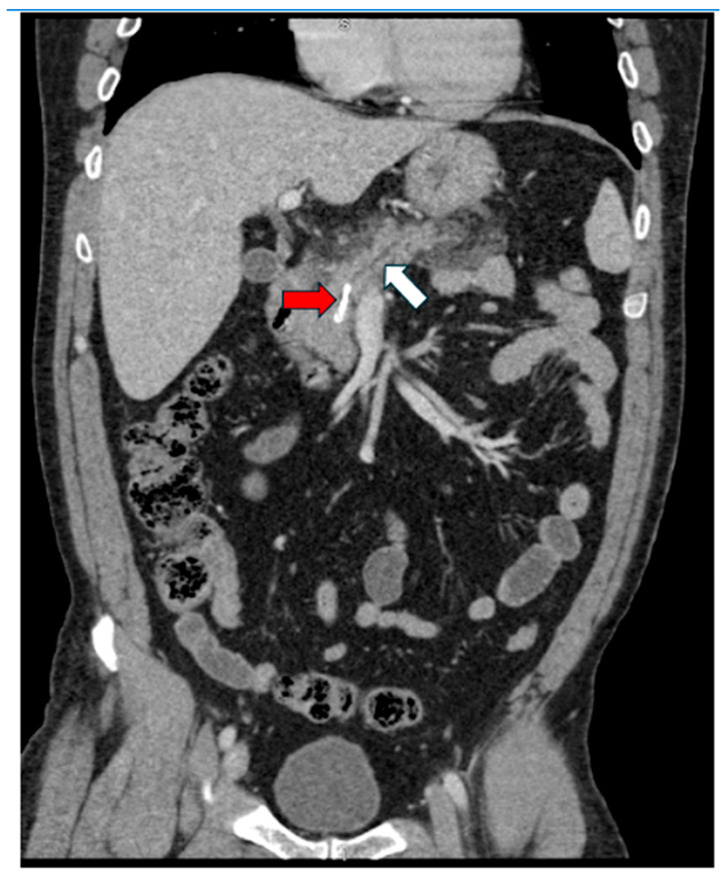
Red arrow points to fragment of stent in the neck of the pancreas. White arrow points to dilated duct upstream.

Twelve patients met the inclusion criteria and underwent endoscopic retrieval for proximally migrated or broken, straight pancreatic duct stents (Figure 1). The mean age was 61.3 years (range, 46–78 years), with a female predominance (58.3%). All patients presented with stents that had migrated proximally into the pancreatic duct, and two patients had fractured stents with retained fragments. All patients were referred after failed removal attempts at external institutions, underscoring the technical difficulty and complexity of these cases.

The stent calibers ranged from 4 French to 7 French, with lengths from 1.5 cm to 12 cm, and the duration of indwelling stents ranged from 4 weeks to over 2 years. In five patients, stents had been in place for more than six months, and in two cases, the stents had exceeded one year. Altogether, the duration from original stent placement to extraction averaged six months across all 12 patients. This prolonged indwelling time likely contributed to the increased difficulty of retrieval, as biofilm formation, epithelial overgrowth, and fibrosis around the stent are more common in chronic stent placements. Among the two patients with fractured stents, one presented with a retained flange in the pancreatic head, and the other had fragmented components in the pancreatic body, posing additional retrieval challenges due to their distal locations.

The most common indication for stent placement was chronic pancreatitis with ductal stricture or pain (*n* = 5), followed by post-surgical leak (*n* = 3), idiopathic ductal obstruction (*n* = 2), and prevention of post-ERCP pancreatitis (*n* = 2). Notably, several of the chronic pancreatitis cases had complex ductal anatomy, including tortuous ducts and upstream dilatation, complicating access and increasing the need for pre-retrieval ductal dilation.

The retrieval technique varied based on the anatomical location and accessibility of the stent. SpyGlass-assisted methods were used when all other methods failed and the pancreatic duct diameter was greater than 4 mm. This method was used in three patients, including one patient with a deeply embedded 5 Fr × 1.5 cm broken stent removed with SpyGlass forceps, and two others in whom a retrieval basket under direct visualization facilitated safe removal. Soehendra retrievers were used in two patients, one with a 5 Fr × 2 cm broken stent retained for over a year, and another with a deeply impacted 7 Fr × 7 cm stent located in the mid-pancreatic duct. In both cases, the device’s ability to engage and drill into the stent proved critical for successful retrieval.

Balloon sweep and balloon dilation with snare retrieval were used in four patients, particularly when ductal narrowing proximal to the stent required dilation to allow device passage. This technique was preferred in cases with limited ductal angulation and more superficial stent positioning. One patient underwent extension pancreatic sphincterotomy to allow deep cannulation and facilitate access to the migrated stent. One patient with a fractured stent flange underwent retrieval using a pediatric biopsy forceps, and another patient with a distal stent lodged near the pancreatic tail required rat-tooth forceps after endoscopic sphincterotomy.

All twelve procedures were completed successfully, yielding a 100% technical success rate. Mean procedural duration was 37.9 min (range, 28–52 min). SpyGlass-assisted retrievals tended to be longer due to the need for cholangioscope setup and navigation. Balloon-based methods were more time-efficient in cases without strictures or when the stent was located in the head or neck of the pancreas. There were no procedural conversions to surgery, and no patients required repeat endoscopy for stent removal failure.

Post-procedural recovery was uneventful in all patients. No major adverse events were observed. Specifically, there were no cases of severe post-ERCP pancreatitis, perforation, gastrointestinal bleeding, or infection. Two patients developed ductal narrowing following stent removal, identified intra-procedurally, and were treated with balloon dilation during the same session. All patients had a temporary pancreatic stent placed post-retrieval to ensure continued drainage and reduce the risk of recurrent obstruction or ductal edema. Three patients experienced mild post-ERCP pancreatitis that resolved quickly with conservative management, consistent with rates reported in the literature for high-risk ERCP cases.

Follow-up ERCP was performed at a median of 3.5 weeks (range, 3–4 weeks), at which time all temporary stents were successfully removed. Ductal patency was confirmed in all patients, with no evidence of recurrent stricture, residual stent fragments, or secondary complications. At the time of follow-up, all patients reported resolution of symptoms, including pain and steatorrhea, and no new episodes of pancreatitis were recorded. No recurrent stent migration or fragmentation was observed during the follow-up period, suggesting a durable procedural outcome.

## 4. Discussion

Recent studies have explored various endoscopic retrieval methods for managing proximally migrated pancreatic duct (PD) stents. Techniques such as balloon sweep, SpyGlass-assisted retrieval, and the use of specialized mechanical devices have become increasingly adopted in clinical practice. A systematic review by Jayanna et al. found that balloon-sweep techniques were effective in retrieving migrated PD stents, with a pooled success rate exceeding 90% [20]. Similarly, the use of SpyGlass systems has been reported to significantly improve visualization and facilitate the retrieval of difficult-to-access or embedded stents, achieving high success rates with minimal complication profiles [21]. However, the SpyGlass system has its limitations—it can only be used in patients with a dilated pancreatic duct measuring greater than 4 mm, which excludes a significant portion of patients from benefiting from this technology [17].

Our study reinforces these observations by demonstrating a 100% technical success rate in retrieving proximally migrated and fractured pancreatic stents across a multicenter cohort. This consistency with previous findings highlights the effectiveness of specialized retrieval tools in expert hands. Importantly, the timing of intervention appears to play a crucial role in minimizing downstream complications. In our study, two patients developed pancreatic duct strictures following delayed retrieval; however, both were successfully managed with balloon dilation and subsequent stenting. These findings are consistent with earlier reports suggesting that delayed intervention increases the risk of ductal injury and chronic complications [22].

The average procedure time in our series (30–45 min) also compares favorably to previous reports, which found that efficient technique selection significantly impacts both patient outcomes and resource utilization. Shorter procedure times not only reduce anesthesia exposure but also contribute to improved turnover rates in endoscopy units, making these techniques more sustainable in high-volume practices. Our experience supports the principle that timely referral to tertiary or quaternary care centers with access to advanced retrieval tools is vital in improving safety and efficacy in these technically challenging cases [23].

**Figure 2:**
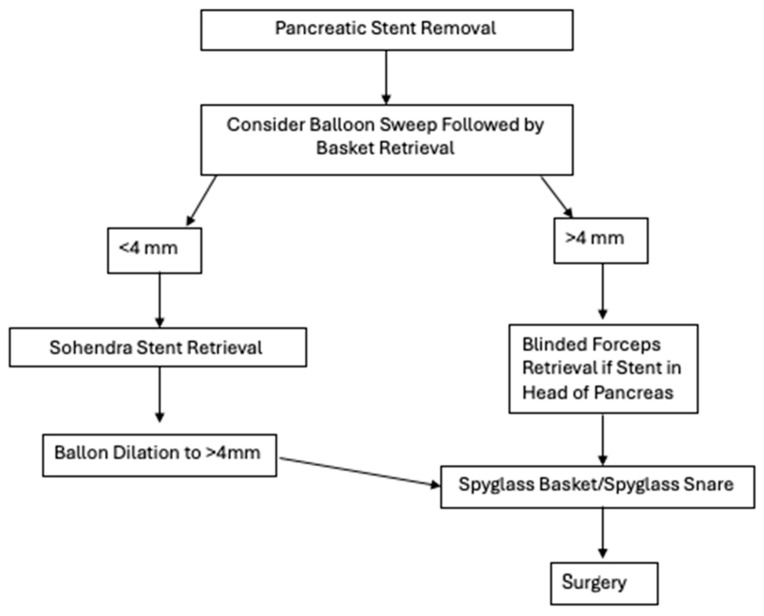
Proposed algorithm on how to successfully retrieve pancreatic stents.

We also proposed a possible algorithmic process to determine how to retrieve pancreatic stents (Figure 2).

This decision-making framework was designed with both ductal anatomy and equipment accessibility in mind and seeks to balance efficacy with invasiveness. The algorithm begins by stratifying patients based on pancreatic duct (PD) diameter—a critical determinant in selecting appropriate retrieval techniques.

For ducts measuring less than 4 mm, initial attempts should favor less-invasive techniques such as balloon sweep followed by basket retrieval. These methods are well-established and can be performed with standard ERCP accessories. However, smaller-caliber ducts present unique challenges, including limited maneuverability and increased risk of mucosal injury. In our algorithm, when standard retrieval fails, we recommend using the Soehendra stent retriever, a corkscrew-like device that is particularly useful for dislodging deeply embedded or fractured stents. The Soehendra retriever’s mechanical advantage lies in its ability to grasp and apply torque, making it especially effective in non-dilated ducts where axial traction is necessary.

If retrieval continues to be unsuccessful in the <4 mm PD subgroup, balloon dilation to >4 mm is advised. This approach not only facilitates potential passage of larger-caliber tools but also permits transition into the same advanced retrieval pathway used for patients with native duct diameters >4 mm. This step is essential in minimizing trauma to the duct wall, which can occur with forceful attempts using inadequately sized instruments.

For ducts already measuring ≥4 mm, balloon sweep and basket retrieval remain appropriate first-line interventions. When these fail, operator judgment becomes essential. If the migrated or fractured stent is located in the head of the pancreas, blinded forceps retrieval may be attempted. Though less commonly used, this technique has shown success in carefully selected patients, particularly when fluoroscopic localization is precise.

When conventional approaches fail, our algorithm prioritizes SpyGlass-assisted retrieval using either SpyGlass baskets or snares. The SpyGlass system provides direct visualization, allowing for controlled and precise retrieval even in cases where stents are partially embedded or surrounded by inflammatory tissue.

If all endoscopic methods fail, our algorithm recommends surgical consultation. Though rarely needed, surgery remains the final recourse in patients with symptomatic, irretrievable stents or those who experience complications such as perforation or refractory pain.

Our proposed algorithm provides a structured yet adaptable framework to guide gastroenterologists through increasingly complex retrieval scenarios. Notably, it emphasizes graduated escalation of intervention based on ductal anatomy, tool availability, and procedural risk.

## 5. Future Research Directions

Despite these encouraging outcomes, several key areas warrant further investigation. Prospective, comparative studies are needed to establish evidence-based algorithms for device selection based on stent type, migration depth, and ductal anatomy. Randomized controlled trials comparing tools such as SpyGlass baskets, Soehendra retrievers, rat-tooth forceps, and novel hybrid devices could provide clearer guidance for real-world practice and optimize patient safety.

Innovations in stent design may help reduce the incidence of migration altogether. Biodegradable pancreatic stents—which gradually disintegrate and eliminate the need for removal—are currently under experimental evaluation [24,25]. These stents may be especially valuable in patients with comorbidities that preclude repeat endoscopy or those with poor follow-up compliance. Additionally, stents with retrievable anti-migration flanges or embedded radiopaque markers could aid in detection and simplify retrieval procedures in the event of migration [26,27]. These enhancements may shorten procedure time and reduce the length of time stents remain undetected within the duct, thereby decreasing the incidence of complications such as infection, stricture, or pancreatitis.

Future work should also explore the integration of artificial intelligence (AI) into endoscopic platforms. AI could assist in real-time decision-making during retrieval attempts by analyzing ductal features, predicting the likelihood of successful retrieval, and suggesting the most appropriate retrieval tool based on prior case data [28]. Incorporating machine learning models into endoscopic imaging systems could enhance procedural precision, reduce operator dependence, and further standardize care.

## 6. Potential Applications

From a clinical standpoint, our data emphasize the importance of routine post-ERCP surveillance, particularly in patients with long-term indwelling stents or altered anatomy. Multidisciplinary case review boards may also aid in the early identification of high-risk patients and facilitate timely intervention planning. In institutions without such systems, the formation of stent management committees or designated “stent surveillance coordinators” may serve as practical solutions.

Additionally, hospital systems should implement automated recall protocols to ensure timely follow-up and stent removal, especially in patients with prolonged indwelling times. Integration with electronic health records (EHRs) could help identify patients due for follow-up imaging or stent exchange, potentially preventing migration before it occurs. Automated alerts, care-pathway templates, and checklists embedded in the EHRs may aid clinicians in closing the loop on stent-related care.

Long-term studies should evaluate the impact of stent retrieval on pancreatic duct integrity, exocrine function, and quality of life. While our short-term follow-up showed excellent resolution of symptoms, the risk of chronic ductal changes or atrophy following repeated interventions remains poorly understood and deserves further study. Furthermore, research into patient-reported outcomes, such as pain, digestive function, and anxiety related to repeated endoscopic procedures, may help better characterize the holistic burden of this complication and guide more patient-centered care strategies.

## Figures and Tables

**Table 1:** Demographic data, stent size, duration of original stent placement, method of stent extraction for each patient.

Patient	Age	Gender	Size of Stent Retrieved	Duration of Original Stent Prior to Extraction	Location	Methodology of Stent Extraction
1	71	M	5 Fr × 1.5 cm (broken stent)	4 weeks	Body	SpyGlass forceps
2	68	M	5 Fr × 5 cm	2 years	Body	SpyGlass basket
3	63	F	5 Fr × 7 cm	8 months	Head	SpyGlass basket
4	67	M	5 Fr × 2 cm (broken stent)	1 year	Tail	Soehendra retriever stent
5	47	F	4 Fr × 5 cm	8 months	Head	Balloon sweep
6	78	M	7 Fr × 5 cm	6 months	Body	Flower basket
7	46	F	7 Fr × 12 cm	3 months	Genu + body	Fractured stent, ext. balloon
8	69	M	7 Fr × 7 cm	4 months	Genu	Balloon dilatation+ snare
9	57	F	7 Fr × 7 cm	4 months	Head + genu	Fractured stent, Soehendra dil + ext. balloon
10	60	F	7 Fr × 7 cm	5 months	Genu	Fractured stent removed balloon dil + ext. balloon
11	46	F	7 Fr × 7 cm	2 months	Head + genu	Fractured stent flange, removed with pediatric biopsy forceps
12	66	M	7 Fr × 12 cm	3 months	Genu + body	Extension pancreatic sphincterotomy+ small rat-tooth forceps

SpyGlass, SpyGlass basket, SpyGlass snare—Boston Scientific Corporation, Marlborough, MA, USA; Soehendra retrievers—Cook Medical, Bloomington, IN, USA; extraction balloons—Boston Scientific Corporation; flower basket—Olympus America, Center Valley, PA, USA.

## Data Availability

Patient consent was waived due to its retrospective nature and the use of de-identified data.

## Abbreviations

The following abbreviations are used in this manuscript:

PS	Pancreatic Stent
ERCP	Endoscopic Retrograde Cholangiopancreatography
PD	Pancreatic Duct
Fr	French (unit of measurement for stents)
Ext. Balloon	Extraction Balloon
Soehendra retriever	Soehendra Stent Retriever
SpyGlass	SpyGlass Endoscopic Visualization System
